# Microbial co-occurrence network demonstrates spatial and climatic trends for global soil diversity

**DOI:** 10.1038/s41597-024-03528-1

**Published:** 2024-06-22

**Authors:** Nikos Pechlivanis, Georgios Karakatsoulis, Konstantinos Kyritsis, Maria Tsagiopoulou, Stefanos Sgardelis, Ilias Kappas, Fotis Psomopoulos

**Affiliations:** 1 Institute of Applied Biosciences, Centre for Research and Technology Hellas, Thermi, 57001 Thessaloniki Greece; 2 Department of Genetics, Development and Molecular Biology, School of Biology, Aristotle University of Thessaloniki, 54124 Thessaloniki, Greece; 3 Centro Nacional de Analisis Genomico (CNAG), C/Baldiri Reixac 4, 08028 Barcelona, Spain; 4 Department of Ecology, School of Biology, Aristotle University of Thessaloniki, 54124 Thessaloniki, Greece

**Keywords:** Network topology, Climate and Earth system modelling, Biodiversity, Statistical methods

## Abstract

Despite recent research efforts to explore the co-occurrence patterns of diverse microbes within soil microbial communities, a substantial knowledge-gap persists regarding global climate influences on soil microbiota behaviour. Comprehending co-occurrence patterns within distinct geoclimatic groups is pivotal for unravelling the ecological structure of microbial communities, that are crucial for preserving ecosystem functions and services. Our study addresses this gap by examining global climatic patterns of microbial diversity. Using data from the Earth Microbiome Project, we analyse a meta-community co-occurrence network for bacterial communities. This method unveils substantial shifts in topological features, highlighting regional and climatic trends. Arid, Polar, and Tropical zones show lower diversity but maintain denser networks, whereas Temperate and Cold zones display higher diversity alongside more modular networks. Furthermore, it identifies significant co-occurrence patterns across diverse climatic regions. Central taxa associated with different climates are pinpointed, highlighting climate’s pivotal role in community structure. In conclusion, our study identifies significant correlations between microbial interactions in diverse climatic regions, contributing valuable insights into the intricate dynamics of soil microbiota.

## Introduction

It is well-established that microbial diversity is important for the environment and human health^1,2^. On a global scale, microorganisms take part in biogeochemical cycles that serve to keep the environment’s numerous elements and compounds, such as sulphur, nitrogen, and carbon, in balance^3,4^. They also aid in soil development, fertility, and the breakdown of organic materials. Soil microbiota provide the primary source of live biomass in soil ecosystems, which is essential to several biogeochemical cycles^5^. A new route for improving soil health through targeted manipulation of the microbiome is being opened up by mounting evidence that climate properties may affect the microbiome^6^. Previous studies revealed that climate change and other anthropogenic impacts must be considered in a systemic context^7^. To this end, the Köppen-Geiger climate classification system^8^ is a widely used method for categorizing the world’s climates based on temperature and precipitation patterns. Developed by Wladimir Köppen and Rudolf Geiger, it assigns a specific code to each climate type (Supplementary Table 1). The major categories include Tropical Climates (designated by letter “*A*” – found near the equator, these climates include tropical rainforests, monsoons, and savannas), Dry Climates (“*B*” – these climates are characterized by low precipitation and include hot deserts, cold deserts, and semi-arid regions), Temperate Climates (“*C*” – these climates are moderate and include humid subtropical, oceanic, and subpolar oceanic climates), Continental Climates (“*D*” – found in the interior of large landmasses, these climates include hot-summer continental and warm-summer continental climates), and Polar climates (“*E*” – these climates are characterized by extremely cold temperatures and are typically found near the Earth’s polar regions). Therefore, to gain valuable insights regarding soil microbiome and develop a systems-level understanding of community function and structure, it is important to characterise soil microbiota, their interactions within different geoclimatic areas, and their contributions to various soil biogeochemical processes.

High-throughput sequencing analysis allows us to characterise the composition and functional characteristics of soil microbial communities over a wide range of geographical scales^1,9^. The majority of microorganisms do not survive in isolation but rather flourish in large-scale communities where they interact closely and provide symbiotic benefits for the group^10,11^. During the development of microbes, these ecological interactions serve as important evolutionary targets for natural selection. It is crucial to comprehend the intricate connections in microbial systems to explain the genetic variability present in microbial populations. The research field of network theory is dedicated to modelling and analysing complex systems comprising interlinked components, or nodes. This approach is essential for gaining a thorough understanding of microbial systems^12,13^. In network theory, nodes are the representation of entities while edges are the representation of their connections and relationships. A special kind of network called a “co-occurrence network” is used to examine co-occurrence patterns in datasets. The objects or entities that co-occur in a particular context are represented by nodes in a co-occurrence network while the frequency or intensity of their links to one another is represented by edges. Microbial co-occurrence networks utilise nodes and edges to represent individual microorganisms and the significant relationships that link them^14^. By analysing these networks, we can evaluate the connections between microorganisms, understand the relationships between them, and identify any co-occurrence patterns they might follow. To this end a variety of network measurements can be used for gaining insight into the linkages and interactions between different microorganisms, the complex interplay within microbial communities, and the ways microbial communities respond to environmental changes or disturbances.

Building microbial co-occurrence networks has proven a challenging process, and as a result, different methods and tools have been utilised for their creation and study. Capturing microbial co-occurrence patterns and identifying their underlying mechanisms can be facilitated through the study of microbial communities on a large scale^6,15,16^. Ecological mechanisms influencing community structure, such as niche filtering and habitat selection, as well as the presence of hub species and possible species interactions can be identified through modules in microbial co-occurrence networks. The Earth Microbiome Project (EMP)^1^, a public database and framework for crowdsourcing sample collection with standardised sequencing and metadata curation, is one example of a huge, complicated microbial community dataset that research teams have investigated^6^. The EMP database provides robust tools for investigating microbial community assembly theories, examining large-scale ecological patterns, and documenting global microbiota. All samples undergo uniform processing protocols and standardization procedures post-collection. Through the application of the EMP Ontology (which is linked to ENVO^17^ and other ontology classes), the database enriches its samples by analysing and categorizing them into habitats, allowing for hierarchical organization that captures essential insights of microbial community diversity (vegetation characteristics, proximity to urban or agricultural environments, sampling from natural reserves, etc.). These samples are predominantly classified into free-living and host-associated microbiota categories. Notably, soil samples represent the largest proportion of the free-living samples distributed worldwide. With a global distribution of over 4,000 free-living soil samples, the database represents a valuable resource for scientific inquiry. Emerging patterns include significant non-random connections, niche specialisation, unanticipated ecological links, and deterministic processes at many taxonomic levels, all of which have not been detected before in co-occurrence data. The properties of co-occurrence patterns at different taxonomic levels and the identification of keystone microbial groups in distinct environments and soils have both been studied effectively using topology-based analysis of huge networks^6,10,18,19^. Furthermore, network-based methodologies have been embraced to ascertain the contribution of microbial dark matter in supporting the Earth and benefiting humanity^15,20^.

The EMP database was used in this study to examine how soil microbiota co-occurred in different successional climatic areas as identified by the Köppen-Geiger climate classification^8^. We aim to identify and investigate topological changes and co-occurrence patterns throughout the successional climatic gradient by analysing co-occurrence networks of soil microbiomes in various climatic zones of the world. This method considers the intricate web of possible interactions between microorganisms in diverse habitats, which helps to contextualise microbial biogeography. Our main objective is to discover and comprehend how the topological characteristics of the co-occurrence network change across various climate zones, which bacterial microbiota patterns are prevalent on Earth, and how soil biodiversity is related to various climatic regions. In line with the Köppen-Geiger climate classification, a network analysis approach was used to evaluate the topological feature dynamics across this continental scale. The current research offers a thorough comprehension of the worldwide topological changes in soil bacterial co-occurrence networks.

## Results

### Distribution of soil samples from the Earth Microbiome Project on a planetary scale

**Fig. 1:**
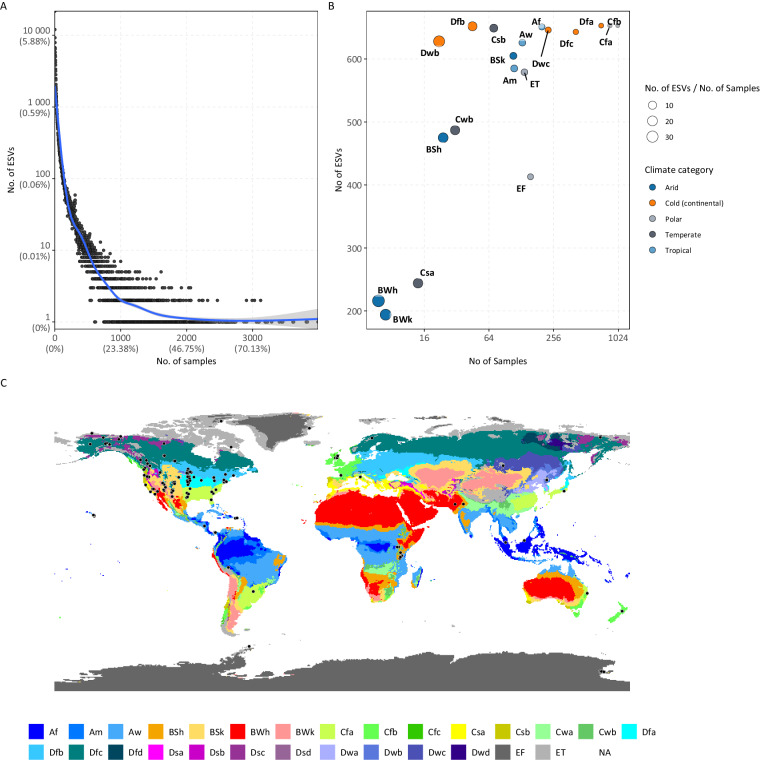
Distribution of samples and ESVs across Köppen-Geiger climate zones. (**A**) Prevalence curves show the increase in the number of consistently present ESVs as the number of samples increases. (**B**) The distribution of samples and ESVs across the five climatic groups and their subcategories based on the Köppen-Geiger climate classification. (**C**) A map of the sample sites with colours representing the different climatic zones.

The quality filtered 90-bp microbiome observation table (release1) was obtained for this experiment from the EMP database. The table included 317,314 Exact Sequence Variants (ESVs) listed in rows, and 23,828 samples organized in columns. In order to limit the analysis only to soil samples, a filter was applied to limit to samples falling under the fourth field of the EMP ontology (EMPO level 3), “Soil (non-saline)” resulting in 4,279 samples and 170,063 ESVs. The dataset included samples coming from 42 studies and located in 19 climatic areas based on the Köppen-Geiger climate classification system. The prevalence curve (Fig. 1A) shows that the bulk (80%) of ESVs were found in less than 8 samples (9%), indicating that the BIOM matrix was sparse. Pre-processing methods were used to filter away the ESVs with low relative abundance present in fewer than 30% of samples in order to reduce noise and false-positive predictions. For more information see “**Materials and Methods**” section. The majority of the ESVs in the filtered BIOM table belonged to Bacteria, while a very minor percentage (below 1%) belonged to Archaea. To preserve just Bacteria ESVs, a last filtering step was applied. The remaining observation table included 4,276 samples and 653 ESVs. The distribution of these samples across all climate zones and across the world is summarised in Fig. 1B,C.

### Diversity analysis (*alpha* and *beta* diversity) reveals geographic and climatic patterns followed across the globe

**Fig. 2:**
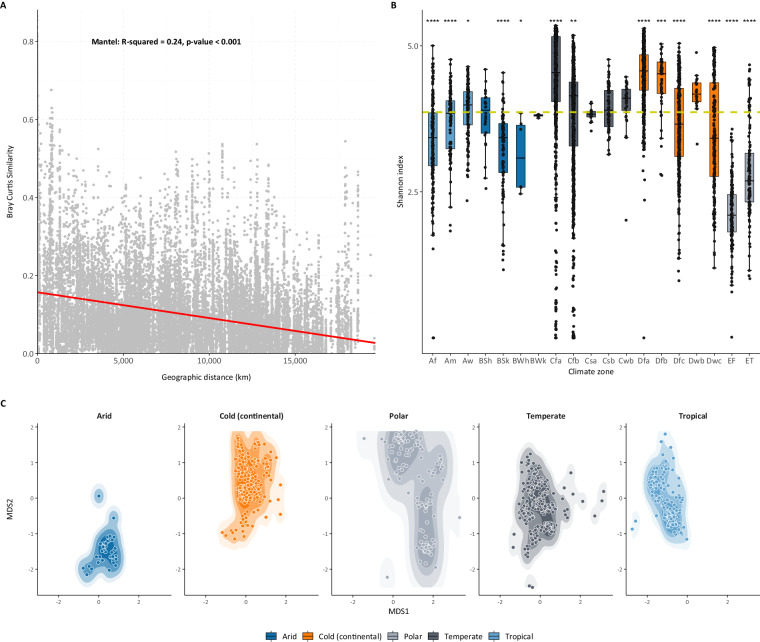
Correlation analysis, alpha diversity, and Non-metric Multidimensional Scaling (NMDS) of samples across climatic zones. (**A**) Correlation analysis showing the relationship between Bray Curtis dissimilarity distance and geographic distance of all samples. (**B**) Boxplots illustrating the within-community (alpha) diversity scores for all zones, with the colour scheme representing the five main climatic groups. (**C**) NMDS plot across individual climatic zones separately.

A Mantel test for the analysis of correlations between Bray Curtis similarity and geographic distance (Fig. 2A), revealed a negative relationship (R-squared = 0.24, p-value < 0.001) indicating that as the geographic distance increases, the similarity tends to decrease. For each sample, within-community (alpha) diversity indices (Shannon, Chao1 γ, and Simpson) were computed. The results (Shannon index in Fig. 2B and Simpson, and Chao1 γ in Supplementary Table 2) are shown as boxplots across all climatic zones. Pairwise comparisons using Holm correction revealed statistically significant differences between the climate categories. Amongst all comparisons, Polar climate zones – *Ice cap* (EF) and *Tundra* (ET) – exhibited notably lower Shannon scores, signifying reduced diversity. Similarly, *Cold semi-arid* (BSk) and *Hot deserts* (BWh) zones also demonstrated lower Shannon scores, indicating a lower diversity in these regions. In contrast, Temperate and Cold zones, excluding *Hot-summer Mediterranean* (Csa), *Subarctic* (Dfc) and *Monsoon-influenced subarctic* (Dwc) zones, yielded higher Shannon scores, suggesting a comparatively greater diversity in these areas. Finally, the Bray-Curtis similarity was used to perform Non-metric Multidimensional Scaling (NMDS), which is shown in Fig. 2C. Permutational multivariate analysis of variance was used to assess the relationship between climatic zone and microbiota composition. The results of permanova (R-squared = 0.19, p-value < 0.01) indicate that climatic regions are correlated with microbiota composition.

### Soil microbial co-occurrence network analysis

The raw count table, as produced from the previous pre-processing and filtering steps, was later on separated into climate specific count tables by maintaining samples and ESVs present in each climatic zone. The SpiecEasi (Sparse and Compositionally Robust Inference of Microbial Ecological Networks)^21^ method was used to build climate specific microbial co-occurrence networks based on the previous observation tables, and resulted in 19 climate-related networks with stability score of 0.05. For all networks, no correlation between abundance and degree was identified (absolute correlation coefficient <0.1), which, along with the presence of a scale-free property (R-squared > 0.77, p-value < 0.05) in all networks, indicates a non-random co-occurrence pattern within the microbial networks, with the majority of ESVs having low-degree values and only a few hub nodes presenting high degree centrality values. The resulted networks included different numbers of nodes and edges. *Hot deserts* (BWh), *Cold desert* (BWk) from the Arid zones and *Hot-summer Mediterranean* (Csa) from the Temperate zone preserved the lowest number of edges to number of nodes ratios (<2). *Subtropical highland or temperate oceanic with dry winters* (Cwb), *Hot semi-arid* (BSh), and *Ice cap* (EF) climates, from Temperate, Arid, and Polar zones respectively produced higher ratios with ~400 nodes and ~2,000 edges. *Tropical monsoon* (Am), *Cold semi-arid* (BSk), *Monsoon-influenced warm-summer humid continental* (Dwb), *Monsoon-influenced subarctic* (Dwc), *Subarctic* (Dfc) and *Tundra* (ET) climates (from Tropical, Arid, Cold, and Polar zones) resulted in even higher ratios with ~580 nodes and ~4.250 edges. Finally, *Tropical rainforest* (Af), *Tropical savanna, wet* (Aw), *Humid subtropical* (Cfa), *Temperate oceanic* (Cfb), *Warm-summer Mediterranean* (Csb), *Hot-summer humid continental* (Dfa), and *Warm-summer humid continental* (Dfb) climates (from Tropical, Temperate, and Cold zones) resulted in the highest ratio scores with ~650 nodes and >5,000 edges. In Supplementary Table 3 a summary is provided with the descriptive results.

### Co-occurrence overrepresentation analysis

**Fig. 3:**
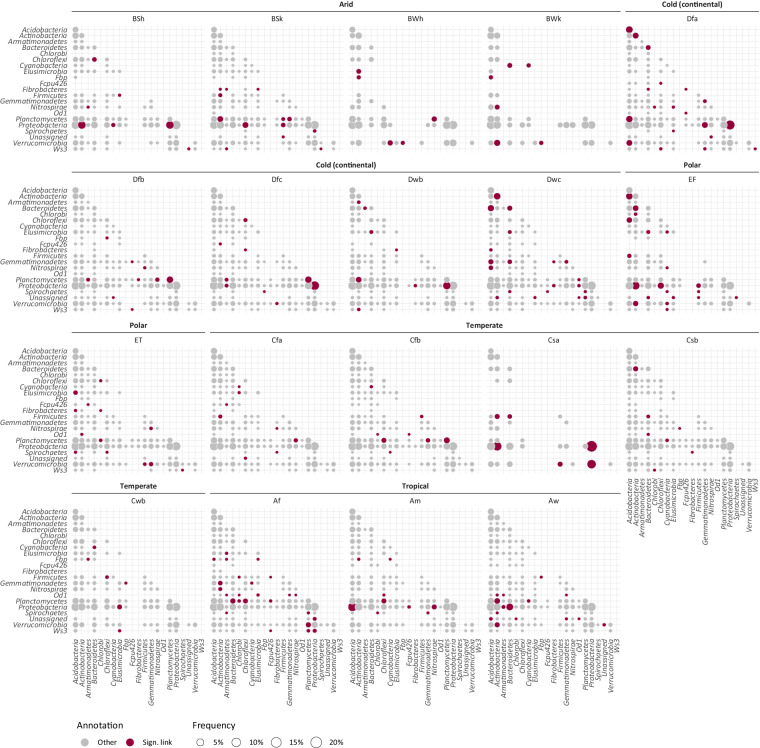
Significant co-occurrence connections among Phylum taxa across diverse climate zones.

The majority of edges were found to be widespread across all analysed climate zones, with approximately 10% (considering the phylum level) identified as climate-specific, existing exclusively in individual climate zones among the 12 investigated. Despite this, multiple taxonomic groupings exhibited overrepresented boundaries across various climate zones, as determined by a significance threshold (p-value < 0.05, detailed in the “**Materials and Methods**” section) compared to random expectations (see Fig. 3). Significant associations were consistently observed across all climate zones, contingent upon the specific taxonomic level under investigation. Starting at the Phylum taxonomic level, significant links were identified in every climate zone, predominantly featuring Acidobacteria, Actinobacteria, Bacteroidetes, Chloroflexi, Planctomycetes, and Proteobacteria.

In the Tropical climate zones (Af, Am, Aw), overrepresented links were noted. The *Tropical rainforest* (Af) zone displayed links between Armatimonadetes, Chlorobi, Elusimicrobia, Fbp, Fcpu426, Od1, Planctomycetes, and Spirochaetes. In the *Tropical monsoon* (Am) climate, links were observed between Acidobacteria, Chloroflexi, Fbp, and Proteobacteria, while the *Tropical savanna, wet* (Aw) zone exhibited links between Actinobacteria, Od1, Proteobacteria, Gemmatimonadetes, and unassigned taxa.

Arid climate zones (BSh, BSk, BWh, BWk) presented overrepresented links. The BSh zone featured significant links with the Proteobacteria, Planctomycetes, and Acidobacteria classes*. Hot semi-arid (BSk)* revealed overrepresented links between Actinobacteria, Fibrobacteres, Firmicutes, and Ws3*. Hot deserts (BWh)* exhibited links between Actinobacteria, Fbp, and Verrucomicrobia, while *Cold desert (BWk)* contained significant links between Actinobacteria, Cyanobacteria, Fbp, and Verrucomicrobia.

In Temperate climate zones (Cfa, Cfb, Csa, Csb, Cwb), overrepresented links were found. *Humid subtropical* (Cfa) included links primarily among Chlorobi and Fibrobacteres, while *Temperate oceanic* (Cfb) contained links between Od1, Planctomycetes. *Hot-summer Mediterranean* (Csa) exhibited links between Actinobacteria, Proteobacteria, and Verrucomicrobia, and *Warm-summer Mediterranean* (Csb) contained links mostly among Bacteroidetes. *Subtropical highland or temperate oceanic with dry winters* (Cwb) included significant links between Elusimicrobia, Fbp, and Ws3.

Cold climate zones revealed a substantial list of overrepresented links. In the *Hot-summer humid continental* (Dfa) climate zone, significant links between Acidobacteria, Bacteroidetes, Chloroflexi, Elusimicrobia, Fcpu426, Gemmatimonadetes, and Ws3 were observed. *Warm-summer humid continental* (Dfb) showed a smaller list of significant links, primarily between Planctomycetes, Fcpu426, and Nitrospirae, while *Subarctic* (Dfc) contained links between Armatimonadetes, Chloroflexi, Planctomycetes, and Proteobacteria. *Monsoon-influenced warm-summer humid continental* (Dwb) included significant links between Actinobacteria, Planctomycetes, and Proteobacteria. *Monsoon-influenced subarctic* (Dwc) featured links between Acidobacteria, Actinobacteria, Bacteroidetes, Fibrobacteres, Od1, and Planctomycetes.

Polar climate zones displayed an extensive list of overrepresented links. The *Ice cap* (EF) zone featured links between Acidobacteria, Actinobacteria, Bacteroidetes, Cyanobacteria, and Firmicutes, while the *Tundra* (ET) zone included links between Acidobacteria, Chlorobi, Verrucomicrobia, and Gemmatimonadetes.

At the Class taxonomic level, the most significant combinations were observed among Acidimicrobiia, Acidobacteriia, Actinobactia, Alphaproteobacteria, Betaproteobacteria, Deltaproteobacteria, Gammaproteobacterial, Anaerolineae, Bacilli, Chloracidobacteria, Chloroflexi, Chloroplast, Chthonomonadetes, Clostridia, Cytophagia, Ellin6529, Elusimicrobia, Fibrobacteria, Fimbriimonadia, and Flavobacteria. As the analysis delved into lower taxonomic levels, additional patterns specific to particular climates became discernible. Notably, significant links were identified between ESVs and unassigned taxa at subsequent taxonomic levels. This finding underscores the potential influence of unidentified species in their respective environments, suggesting their crucial role in shaping ecological dynamics. For a detailed listing of all overrepresented linkages according to taxonomic rankings, please refer to Supplementary Table 4.

### Global network properties unveil climate-related community patterns

**Fig. 4:**
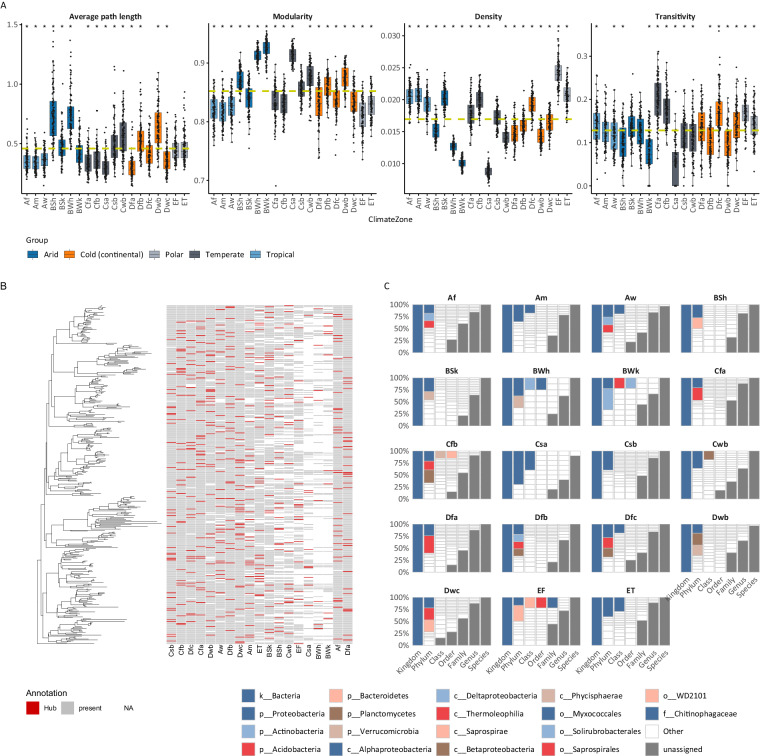
Co-occurrence network analysis across different climatic zones. (**A**) Boxplots depicting global network properties (average path length, modularity, density, and transitivity) for each of the fifteen subnetworks across all climate zones, with colours representing the five main climatic groups. (**B**) A phylogenetic tree showing the 83 microbial species identified as hubs in at least one climate zone, with a heatmap indicating their presence (grey), absence (white), or hub identification (red). (**C**) Taxonomic barplots summarising the taxonomy of the identified hubs across all climate zones.

The climate-related subnetworks were compared based on their global network features, using four criteria: transitivity, network density, modularity (based on fast greedy clustering), and average path length. Pairwise comparisons between all climatic zones revealed substantial discrepancies. In terms of network density, Polar zones exhibited denser networks, while *Hot deserts* (BWh), *Cold deserts* (BWk), and *Hot-summer Mediterranean* (Csa) zones displayed lower density scores. Additionally, *Cold semi-arid* (BSk) and *Hot-summer Mediterranean* (Csa) climate zones yielded higher and lower density scores, respectively, compared to their respective climate groups. Similar patterns were observed in modularity scores, with most climate groups exhibiting low values, while BSk and Csa zones distinctly followed different distributions than the rest of their respective category. Certain climate zones, specifically *Hot semi-arid* (BSh) and *Hot deserts* (BWh) from the Arid zones, *Warm-summer Mediterranean* (Csb) and *Subtropical highland or temperate oceanic with dry winters* (Cwb) from the Temperate zones, and *Warm-summer humid continental* (Dfb) and *Monsoon-influenced warm-summer humid continental* (Dwb) from the Cold zones, resulted in high average path length scores. Notably, *Hot-summer Mediterranean* (Csa) zone exhibited the lowest scores in terms of transitivity. The results, depicted in Fig. 4A, highlight the diverse and distinctive network characteristics across various climatic zones, underscoring the complexity and uniqueness of microbial interactions within these environments.

### Topological properties

Within the relevant subnetworks^22^, significant variation in topological features, including degree, betweenness, and eigenvector centralities, was observed. Specifically, *Hot deserts* (BWh), *Cold desert* (BWk), and *Hot-summer Mediterranean* (Csa) zones exhibited the lowest degree and eigenvector centrality, along with lower betweenness centrality values. It’s worth noting that *Hot semi-arid* (BSk) from the Arid zone and *Hot-summer Mediterranean* (Csa) from the Temperate zones appeared to deviate from the trends observed in the other zones within their respective climate groups. These distinctive patterns in network characteristics underscore the heterogeneity of microbial interactions and connectivity within different climatic zones. The unique behaviors of BSk and Csa zones within their climate groups suggest nuanced ecological dynamics that contribute to the overall complexity of microbial networks in these specific environments.

### Hub taxa

A total of 500 hub nodes were identified across diverse climate zones, as illustrated in Fig. 4B,C. These hubs predominantly represent taxonomic phyla such as Acidobacteria, Actinobacteria, Bacteroidetes, Chloroflexi, Gemmatimonadetes, Nitrospirae, Planctomycetes, Proteobacteria, and Verrucomicrobia. In tropical zones (Af, Am, Aw) 93 hub nodes were identified, primarily composed of Alphaproteobacteria, Betaproteobacteria, Thermoleophilia, Acidobacteria, and Actinobacteria. Arid zones (BSh, BSk, BWh, BWk) yielded 68 hub nodes, with a predominant presence of deltaproteobacteria. Temperate zones (Cfa, Cfb, Cfc, Csb, Csc, Cwb) displayed 135 hub nodes, mainly featuring Alphaproteobacterial, Betaproteobacteria, Deltaproteobacteria, and Actinobacteria. Cold zones revealed 163 hubs, representing Alphaproteobacteria, Betaproteobacteria, Acidobacteria, Planctomycetia, and Nitrospira. Polar zones (EF, ET) comprised 46 hub nodes, with Saprospirae and Betaproteobacteria prevalent in *Ice cap* (EF), and Alphaproteobacterial and Acidimicrobia in *Tundra* (ET). It is noteworthy that certain hub nodes are present in multiple climatic zones but are recognized as hubs only in specific regions. The majority of hubs, as depicted in the hub presentation, belong to unassigned taxa at the genus level. Among the climatic zones, *Hot deserts* (BWh), *Cold desert* (BWk), and *Hot-summer Mediterranean* (Csa) exhibited a lower number of hubs. In Supplementary Table 5 a comprehensive list of all identified hubs in each analyzed subset is provided.

### Negative co-occurrence links

In diverse climate-related sub-networks, the application of the SpiecEasi method has facilitated the identification of negative co-occurrence edges among bacterial taxa^22^. Across all examined sub-networks, the percentage of negative edges fell within the range of 20% to 40%, indicating the presence of negative co-occurrence interactions within these bacterial communities. Notably, *Cold desert* (BWk) and *Hot-summer Mediterranean* (Csa) climate zones stood out with percentages of negative links exceeding 40% (44% and 45%, respectively). These negative edges were predominantly associated with the phyla Acidobacteria, Actinobacteria, Proteobacteria, and unassigned ESVs. A compelling observation is that the majority of sub-networks featured more than 10% negative edges, suggesting that these unfavorable connections are widespread in bacterial communities. This discovery of negative co-occurrence edges provides valuable insights into the intricate connections between bacterial species in various habitats. Understanding the dynamics of bacterial populations in different climatic zones and their responses to environmental changes can be significantly enhanced by considering these findings.

### Consistency and reproducibility

To ensure result repeatability, a more stringent filter of 0.01% was applied to the relative abundance of ESVs, as detailed in the “**Materials and Methods**” section^22^. This adjustment led to a reduced number of ESVs per climate zone. However, the observed patterns remained consistent. Regarding diversity, the Polar zones exhibited the lowest Shannon index scores, followed by the arid zones of *Hot semi-arid* (BSk) and *Hot deserts* (BWh). Microbial composition exhibited a strong correlation with climatic regions. Utilizing the SpiecEasi algorithm on 19 climate-related microbial abundance tables resulted in the creation of 19 scale-free microbial co-occurrence networks. Zones such as *Hot deserts* (BWh), *Cold desert* (BWk), and *Hot-summer Mediterranean* (Csa) displayed both the lowest number of nodes and edges. In microbial over-representation analysis, fewer significant connections were identified, yet consistent trends were observed. Dominant phyla included Acidobacteria, Actinobacteria, Proteobacteria, Chloroflexi, and Bacteroidetes. Additionally, a more focused list of hub taxa (238 hub nodes) was identified, with dominance by Acidobacteria, Actinobacteria, Proteobacteria, and Planctomycetes. Examining global network properties revealed consistent patterns, with arid zones *Hot deserts* (BWh) and *Cold desert* (BWk) displaying less dense networks. Notably, *Cold desert* (BWk) exhibited a much less modular network structure. These findings reinforce the robustness and consistency of the observed microbial patterns across various climatic zones.

## Discussion

Our exploration of microbial interactions in diverse climatic zones has encompassed both taxonomic and network-based analyses, shedding light on the intricate dynamics shaping bacterial communities. In order to effectively demonstrate the developed framework, we utilized the largest available microbial dataset through EMP of free-living organisms (i.e. not host-related). To this end, more than 4,000 soil samples distributed globally have been analysed. Initially, the Mantel test revealed a negative correlation (R-squared = 0.24, p-value < 0.001), between Bray Curtis similarity and geographic distance, indicating that as distance increases, microbial similarity decreases. This suggests distinct microbial compositions across geographical locations. On the contrary, Non-metric Multidimensional Scaling analysis showed that climate zones significantly influence microbiota composition variations (permanova R-squared = 0.19, p-value < 0.01). This finding aligns with the concept of distance-decay in microbial communities, emphasizing the influence of geoclimatic factors on community structure, as also supported by previous works^23,24^. Furthermore, the within-community diversity indices (Shannon, Chao1 γ, and Simpson) exhibited significant variations across different climates. On one hand, Polar zones (EF and ET) and Arid zones (BSk and BWh) displayed lower Shannon scores, indicating reduced microbial diversity in these extreme environments. Conversely, Temperate and Cold zones (excluding Csa, Dfc, and Dwc zones) exhibited higher Shannon scores, suggesting a comparatively greater diversity in these regions.

Additional insights into the structural aspects of microbial communities were provided from the subsequent creation of climate-specific microbial co-occurrence networks using the SpiecEasi algorithm. The resulted microbial co-occurrence networks exhibit a scale-free structure, similar to other microbiome co-occurrence networks^16,18,25,26^ or real-world networks like the World Wide Web^27^. The presence of a scale-free characteristic in the networks implies the existence of a limited number of highly connected nodes, referred to as hub taxa, with the majority having relatively fewer connections. This scale-free network structure suggests the formation of an ultra-small world network, underscoring the pivotal role of microbial interaction relationships in shaping the assembly processes of microbial communities. Despite variations in network size, certain climates, such as *Hot deserts* (BWh), *Cold desert* (BWk), and *Hot-summer Mediterranean* (Csa), exhibited lower ratios of edges to nodes, indicating a more constrained and interconnected network structure. In contrast, climates such as *Subtropical highland or temperate oceanic with dry winters* (Cwb), *Hot semi-arid* (BSh), and *Ice cap* (EF) displayed higher ratios, signifying more intricate and interconnected microbial networks.

To ensure that essential trends are captured, four independent metrics —average path length, network density, modularity based on fast greedy clustering, and transitivity— were used to study climate-related co-occurrence networks of microbial communities. In particular, Polar zones (EF, ET) exhibited high-density networks, though with lower modularity and average path length scores compared to other subnetworks. Τhese findings are in agreement and further supported by previous studies which indicate a strong link between network modularity and the corresponding environment^6,18^. Reduced modularity may imply higher vulnerability to disturbances in these microbial communities, potentially due to decreased redundancy and buffering capacity. In our study the network structure is more modular in *Hot deserts* (BWh), *Cold desert* (BWk), and *Hot-summer Mediterranean* (Csa) climate areas than in other temperature zones, which shows that some groups of microbial species are closely linked and co-occur often within these particular climatic zones. This may also suggest that some microbial species or groups are more important or influential in these climatic zones and are intricately linked to one another. In addition, considering that modules in microbial co-occurrence networks potentially represent distinct ecological niches^11^, the current distribution of ESVs within the respective geoclimatic regions can provide insights into the similarity of microbial co-occurrence patterns across diverse climates. Regarding the average path length, higher scores are observed in *Hot semi-arid* (BSh) and *Hot deserts* (BWh) indicating a complex and potentially modular community structure. This may imply dependencies mediated by other microorganisms, providing insights into the ecological complexity and functional redundancy within the microbial ecosystem. It is noteworthy to highlight that distinct climate zones exhibit variations in their distribution patterns compared to their respective climate groups. Specifically, the *Cold semi-arid* (BSk) climate areas display trends that deviate entirely from those observed in the remaining areas within the Arid climate zone. As suggested by previous works^28,29^, investigating these variations is crucial for various sectors, including agriculture, water management, conservation, urban planning, and disaster risk reduction.

Furthermore, the direct relationships between ESVs in microbial communities were also examined across different climate zones, with several taxonomic boundaries being overrepresented in specific climatic zones. An interesting observation is that a considerable portion of the observed ESVs were present in multiple climate zones, but they only formed co-occurrence links within specific climate environments. This highlights the critical influence of climate on community structure, aligning with previous findings where specific taxa formed links within distinct environments^6,18^. Prior studies have also identified variations in both taxonomic and functional diversity across different geographic locations, influenced by soil characteristics such as soil class and pH levels^18,30^. Even more climate-specific patterns are observed at lower taxonomic levels, the majority of which comprise unassigned species.

Additionally, hub nodes in various climatic zones were identified. The taxonomic classes Alphaproteobacteria, Betaproteobacteria, Gammaproteobacteria, Deltaproteobacteria, and Actinobacteria controlled these hubs to a large extent. Most of these hub nodes were associated with the Phyla Acidobacteria, Actinobacteria, Bacteroidetes, and Chloroflexi. Given that these hubs acquired edges with various taxonomic profiles in different environments, those taxa may have the potential to synchronize ecological processes over broad ecosystems. These results may have repercussions for our comprehension of the composition and operation of microbial communities in various temperate zones, as well as their potential responses to environmental disturbances or modifications^31^. They also emphasise the significance of considering the intricate relationships that exist between microbial species and their surroundings, since these relationships may eventually have an impact on ecosystem processes and human health^32–34^. Together these findings suggest that microbial interactions may have a more substantial impact on soil functions than species diversity, underscoring the significance of comprehending the mechanisms that shape microbial communities in distinct regions and climates.

In conclusion, this work offers a thorough investigation of the patterns of microbial co-occurrence across the globe in connection with the system of planetary climate classification^8^. We discovered interconnection patterns in the Earth’s microbial co-occurrence network that span climate-specific environments, and we propose that these patterns are important determinants of microbial community characteristics that can be used in conjunction with microbial taxon compositional profiles. It is important to highlight that the majority of microbial co-occurrence connections presented in this study lack experimental validation, which is crucial for establishing the credibility of the results. While co-occurrence network analysis provides valuable insights, experimental confirmation is necessary to ensure the accuracy and reliability of the findings. An additional area warranting exploration is the geographical scope of our study. Even though we have established a significant correlation between climate and soil diversity, our analysis primarily drew from samples in North America and Europe. This representation excludes certain climatic regions, necessitating further investigation into areas like Africa, Australia, and Asia. However, it is essential to acknowledge that expanding the reference catalogue to encompass a broader range of microbial geoclimatic patterns requires substantial time and effort at both the academic and government levels. Moreover, it is essential to conduct further investigations into the functional properties and biological roles of microbiome communities within each environment^35^. Understanding the specific functions and contributions of these communities in their respective environments will provide a more comprehensive understanding of their ecological significance and potential implications for various ecosystems. It is also important to investigate how additional factors influence the biodiversity of a given area, as noted by previous works^30,36,37^. Finally, the present study mainly focused on bacterial communities at the EMPO3 “Soil (non-saline)” level. Therefore, in order to thoroughly decipher patterns of global microbial co-occurrence, additional research is required to fill in the gaps for other communities and EMPO levels within the EMP framework, or even expand beyond the scope of the EMP database. It is particularly intriguing that novel techniques need to be developed in order to investigate the planet’s biodiversity in relation to forthcoming climate changes^8,38^.

## Materials and methods

### Data retrieval

The Earth Microbiome Project has produced a variety of microbiome datasets that equally reflect various settings (EMPO level 3) and investigations conducted. These datasets are based on sequence data from the EMP database that have been corrected for errors and trimmed using Deblur in Qiime1. The complete workflow for soil collection, metadata curation, DNA extraction, sequencing, and sequence preparation, as well as the various subsets, can be retrieved from the EMP website (or FTP site). The complete quality filtered 90-bp BIOM table of release1 samples was downloaded from the FTP site (http://ftp.microbio.me/emp/release1/) and used for the purposes of this study. The table encompasses all ESV observations supplied by EMP and has been previously investigated by other research endeavours^1^. Additionally, only samples that fit the EMPO3 ontology “Soil (non-saline)” were selected for analysis due to their simplicity. These samples were classified and labelled with climatic zones in line with the Köppen-Geiger climate classification system, based on the geographic coordinates of each location. In Supplementary Table 6, a comprehensive list is provided of all samples and accession numbers for published data that have been used in this study. R (v4.3.1) was used for all data processing, statistical analysis, and network analysis. The rbiom package was used to handle BIOM tables while the data.table and stringr packages were used to analyse the data. The ggplot2 package was used for all visualisations.

### ESV/Sample pre-processing

Several pre-processing steps were applied to ensure resilience in the analysis. To prevent the introduction of noise in the data and avoid false positive observations, ESVs with a relative abundance of less than 0.001% and present in fewer than 30% of samples were removed. It should be noted that this filter was chosen as filtering to 30% of samples in each climatic region led to too few ESVs, even none in some zones. This resulted in false positive predictions during network construction due to low stability scores. Ultimately, filtering all samples produced networks with stability scores closer to the desired threshold (~0.05) while retaining dominant taxa. Additionally, a stricter filtering threshold of 0.01% was employed to assess its impact on the analysis. In the “**Results**” section, we present the results for the first filtering procedure unless otherwise indicated. Since Archaea were underrepresented, only Bacteria were included in the analysis. Finally, to ensure sufficient sample representation for each climatic zone, a filter was applied to retain only climate zones with at least five samples.

### Alpha and beta diversity analyses

Firstly, in order to examine the effect of geographic distance on the bacterial communities, a distance decay of community similarity analysis was carried out using the Vincenty (ellipsoid) method for the geographic distance calculation and the Mantel correlation test. In addition, the “vegan” package was used to estimate the alpha and beta diversity measures. Three indices — Shannon, Chao1 γ, and Simpson — were employed to measure the alpha diversity. Wilcoxon signed-rank tests were used to compare the corresponding climatic zones in pairs. The Holm technique was used to correct p-values. The Bray-Curtis dissimilarity distance was used as the basis for the Non-metric Multidimensional Scaling, to provide a graphical representation of community differentiation on the NMDS two first axes plane as an indication of beta diversity. Using the “adonis2” function from the “vegan” package, permutational multivariate analysis was carried out to evaluate the relationship between climatic zones and microbiota composition.

### Co-occurrence network inference

We built several networks of microbial co-occurrences, with nodes representing ESVs and edges representing direct co-occurrence relationships between ESVs. We correlated each one of them with information on the classification of the world’s climate, based on the Köppen-Geiger system.

### Network (and sub-networks) construction

First, the whole abundance table generated for the earlier analysis, was processed and separated into several climate specific abundance tables by reserving each time the samples and ESVs that were present in each climate zone. For the construction of microbial co-occurrence networks we used the SpiecEasi (Sparse and Compositionally Robust Inference of Microbial Ecological Networks)^21^ method which was applied to account for the sparsity and compositionality that the metagenomic datasets possess. The algorithm derives the conditional independence structure from the data using graphical models. The program used the raw abundance table as input and calculated the conditional dependency of each pair of ESVs using the neighbourhood selection of the MB (Meinshausen and Bühlmann). In order to select the best sparsity parameter, the Stability Approach to Regularization Selection (StARS) variability (i.e., minimum) threshold was set to 0.01 for all networks. This was done using the StARS approach. The SpiecEasi algorithm was applied to every climate – related abundance table previously generated. The resulted networks were then turned into an “igraph” object for further analysis. In order to examine the scale-free property, we applied a polynomial regression, having as independent variable the degree centralities and as dependent its relative frequency in the network.

### Network bootstrap analysis

A bootstrap approach was employed by establishing a random set of sub-networks with the same number of nodes to make sure that changes in network features were not solely due to the number of nodes that each climate-specific network includes. More precisely, a loop of 100 iterations was developed while retaining the same number of random nodes (along with the connections they form) that were picked for each of the climate zone network.

### Network analysis

#### Overrepresentation analysis

Using the hypergeometric distribution, taxon-taxon counts at all taxonomic ranks were evaluated for overrepresentation significance. The Holm technique was used for adjusting the p-values. Taxon-taxon links with adjusted p-values lower than 0.05 were considered as overrepresented.

#### Global Properties

The igraph package was used to determine global network characteristics. In particular, transitivity, density, modularity, and average path length were examined. Modularity was evaluated by the fast-greedy clustering approach. Based on the aforementioned bootstrap methodology, each statistic was calculated 100 times. Wilcoxon signed-rank tests were used to compare the corresponding climatic zones in pairs. The Holm technique was used to correct p-values.

#### Topological features

Topological characteristics at the node level, such as degree, betweenness, closeness, and eigenvector centralities, were calculated using the igraph package. In order to consider the number of nodes within each subnetwork, we determined the average centrality metric value for every node in the respective subnetwork. Wilcoxon signed-rank tests were used to evaluate pairwise differences of the topological characteristics between climatic zones. The Holm technique was used to correct p-values.

#### Hub (Key ESVs) identification

The empirical 95% quantile of all degree centralities in the relevant network was used to identify hub nodes as nodes with degree centrality values higher than that.

### Supplementary information

Supplementary Table 2

Supplementary Table 3

Supplementary Table 4

Supplementary Table 5

Supplementary Table 6

Supplementary Table 1

## Data Availability

EMP observation tables, metadata, and other data that were used for the present study can be accessed from the FTP site (http://ftp.microbio.me/emp/release1). All processed data and results (organized in their respective folders along with the relevant documentation) can be accessed from 10.5281/zenodo.10650154^22^.

## Code availability

All R scripts used in this study and any other analysis files are available at https://github.com/npechl/empclima. Complete documentation and results files for each analysis conducted in this project are also provided at https://github.com/npechl/empclima.
